# Protecting Intestinal Epithelial Cell Number 6 against Fission Neutron Irradiation through NF-*κ*B Signaling Pathway

**DOI:** 10.1155/2015/124721

**Published:** 2015-03-19

**Authors:** Gong-Min Chang, Ya-Bing Gao, Shui-Ming Wang, Xin-Ping Xu, Li Zhao, Jing Zhang, Jin-Feng Li, Yun-Liang Wang, Rui-Yun Peng

**Affiliations:** ^1^Department of Experimental Pathology, Beijing Institute of Radiation Medicine, 27 Taiping Road, Haidian District 100850, China; ^2^Department of Medical Oncology, Air Force PLA General Hospital, Fucheng Road No. 30, Haidian District, Beijing 100142, China; ^3^The Neurology Department of the 148th Hospital, 20 Zhanbei Road, Zibo 255300, China

## Abstract

The purpose of this paper is to explore the change of NF-*κ*B signaling pathway in intestinal epithelial cell induced by fission neutron irradiation and the influence of the PI3K/Akt pathway inhibitor LY294002. Three groups of IEC-6 cell lines were given: control group, neutron irradiation of 4Gy group, and neutron irradiation of 4Gy with LY294002 treatment group. Except the control group, the other groups were irradiated by neutron of 4Gy. LY294002 was given before 24 hours of neutron irradiation. At 6 h and 24 h after neutron irradiation, the morphologic changes, proliferation ability, apoptosis, and necrosis rates of the IEC-6 cell lines were assayed and the changes of NF-*κ*B and PI3K/Akt pathway were detected. At 6 h and 24 h after neutron irradiation of 4Gy, the proliferation ability of the IEC-6 cells decreased and lots of apoptotic and necrotic cells were found. The injuries in LY294002 treatment and neutron irradiation group were more serious than those in control and neutron irradiation groups. The results suggest that IEC-6 cells were obviously damaged and induced serious apoptosis and necrosis by neutron irradiation of 4Gy; the NF-*κ*B signaling pathway in IEC-6 was activated by neutron irradiation which could protect IEC-6 against injury by neutron irradiation; LY294002 could inhibit the activity of IEC-6 cells.

## 1. Introduction

As we all know, the pathological change of intestine induced by neutron irradiation is on the whole elucidated, while the mechanisms of injury were not elucidated completely. The transcription factor nuclear factor-kappa B (NF-*κ*B) plays a pivotal role in the cellular response to various kinds of stress situations. Exposure to extracellular stimuli, such as microbial products and proinflammatory cytokines, as well as internally initiated stress signals derived from reactive oxygen species, hypoxia, or endoplasmatic reticulum stress, initiates signaling pathways that activate the gene expression-inducing capacity of NF-*κ*B [1]. On the one hand, NF-*κ*B plays an important role in inflammatory reaction; on the other hand, NF-*κ*B can protect intestinal epithelial cell against damage [2]. NF-*κ*B is composed of p65 and p50 subunit. NF-*κ*B combines together with the inhibitor of kappa B (I*κ*B) family in the cytoplasm which is not activity. Many extracellular stimulation can cause a series of cascade reactions of intracell. I*κ*B kinase (IKK) family proteins activate I*κ*B family proteins segregating NF-*κ*B and I*κ*B family proteins; then, NF-*κ*B is activated and controls many genes transcription [3, 4]. LY294002 is the classic inhibitor of phosphatidylinositol-3-kinase (PI3K). PI3K and Akt can induce NF-*κ*B activation. LY294002 inhibits the activation of PI3K/Akt which can inhibit the activation of NF-*κ*B at the same time [5]. We established intestinal epithelial cell model injured by neutron irradiation of 4Gy, to study the protection of NF-*κ*B signaling pathway on intestinal epithelial cell injured by neutron irradiation and explore how PI3K/Akt regulate NF-*κ*B signaling pathway. Our studies might provide important theoretical and practical evidence about intestine injured by neutron irradiation.

## 2. Materials and Methods

### 2.1. Cell Culture and Reagents

Intestinal epithelial cell number 6 (IEC-6) cell lines (origin of SD rat) were kindly provided by Professor Qingliang Luo. IEC-6 cells were inoculated in Dulbecco's modified eagle medium (DMEM) (Sigma-Aldrich Company, New Jersey, USA). The media were supplemented with 10% fetal bovine serum (FBS) (Yuan Heng Sheng Ma Biology Technology Research Institute, Beijing, China). The culture solutions were replaced every two days and IEC-6 cells were subcultured by trypsinization every three days. Three groups were randomly given: control group, neutron irradiation group, and LY294002 (Cell Signaling Technology Company, Beverly, MA, USA) treatment group. LY294002 was purchased from Cell Signaling Technology and 40 millimolar (mM) stock solutions of LY294002 in dimethyl sulfoxide (DMSO) (Beijing Chemical Reagent Factory, China) were stored at −20°C. LY294002 was added into the culture solutions 24 hours before neutron irradiation (final LY294002 concentration was 10 *μ*M). The control groups received isovolumic DMSO. Annexin V fluorescein isothiocyanate (Annexin V-FITC) kit was from Beijing Bao Sai Biotech of China. Anti-NF-*κ*B and anti-phosphor-NF-*κ*B, anti-IKK*α*/*β* and anti-phosphor-IKK*α*/*β*, and anti-I*κ*B*α* and anti-phosphor-*κ*IB*α* antibodies were purchased from Cell Signaling Technology Company of America (NF-*κ*B Pathway Sampler Kit, including primary antibodies and secondary antibodies, all antibodies were stored at–20°C). Anti-PI3K and anti-phosphor-PI3K, anti-Akt and anti-phosphor-Akt antibodies were also purchased from Cell Signaling Technology Company of America. All primary antibodies were diluted to 1 : 1000 in 0.1% Tween in* tris*-buffered saline (TBS-T). Secondary antibodies were diluted to 1 : 5000 in 0.1% TBS-T. Anti-glyceraldehyde-3-phosphate dehydrogenase (GAPDH) was gotten from KangChen Biotechnology of China (stored at 4°C). Anti-GAPDH was diluted to 1 : 10 000 in 0.1% TBS-T.

### 2.2. Neutron Irradiation

Fission neutron source was provided by Nuclear Energy Technology Design Academy of Tsinghua University, Beijing, China. The power of the reactor is 50 kilowatt (kW). The rate of neutron and *γ* ray is 9 : 1 (neutron occupies 90%). The average energy of neutron was 1.33 MeV. The dose rate of neutron was 39.04cGy/min and its absorbed dose was 4Gy. The IEC-6 cell culture bottles were fixed on a plastic disc. When the IEC-6 cell culture bottles were radiated by neutron, the plastic disc was rotating (rotation speed of 10 cycles per minute) in order to assure the IEC-6 cells received even neutron irradiation.

### 2.3. Inverted Phase Contrast Microscope (IPCM) Assay

The morphologic changes of the IEC-6 cells were observed by IPCM (Olympus, Japan) at 6 h and 24 h after neutron irradiation.

### 2.4. 3-(4,5-Dimethylthiazol-2-yl)-2,5-diphenyltetrazolium Bromide (MTT) Assay

IEC-6 cells were inoculated in 96-pore plate (cell density 3~5 ×10^4^/mL, 200 *μ*L/pore). The IEC-6 cells were assayed by MTT (Gibco, USA) colorimetry at 6 h and 24 h after neutron irradiation of 4Gy. Experiment procedure: (1) add 20 *μ*L MTT solution into each pore of the 96-pore plate; (2) put the 96-pore plate into 37°C attemperator for 4 h; (3) suck and discard the supernatant and add 200 *μ*L DMSO into each pore of the 96-pore plate; (4) fix the 96-pore plate onto a shaker and shake it thoroughly so that the crystallizations were dissolved completely; (5) assay the optical density (OD) value of each pore by means of an enzyme linked immunosorbent assay detector (wavelength of 570 nm).

### 2.5. Flow Cytometry (FCM) Assay

The apoptosis and necrosis rates of the IEC-6 cells were assayed at 6 h and 24 h after neutron irradiation of 4Gy. Experiment procedure according to Annexin V-FITC kit (Bao Sai Biology Technology Company, Beijing, China) instruction: (1) trypsinize the IEC-6 cells by 0.25% trypsin and wash them at 4°C 0.1% phosphate buffered solution (PBS) by centrifuge; (2) modulate the IEC-6 cells concentration to 5 × 10^5^~1 × 10^6^/mL and wash them at 4°C 0.1% PBS by centrifuge again; (3) suspend the IEC-6 cells in 200 *μ*L buffer solution; (4) add 10 mL Annexin V-FITC and 5 mL propidium iodide (PI) into the buffer solution; (5) admix above-mentioned solution uniformly and leave the solution to react at the room or 4°C temperature in the dark; (6) add 300 *μ*L binding buffer into above-mentioned solution and then assay the apoptosis and necrosis rates of the IEC-6 cells by FCM (B-D, America).

### 2.6. Western Blotting Assay

IEC-6 cells were collected and the total proteins were extracted at 6 h and 24 h after neutron irradiation of 4Gy. Proteins were extracted using a Whole Cell Extraction Kit (EMD Millipore Corporation, Billerica, MA, USA), according to the instructions. The proteins were separated in the sodium dodecyl sulfate polyacrylamide gel electrophoresis (SDS-PAGE) and transferred to the polyvinylidene difluoride (PVDF) membrane (EMD Millipore Corporation, Billerica, MA, USA). We judged the interest protein straps according to the protein marker. The PVDF membranes were blocked in 5% nonfat milk (diluted in 0.1% TBS-T) for 2 h at room temperature. The PVDF membranes were probed with primary antibodies on the rocking bed overnight at 4°C, washed in 0.1% TBS-T three times of 10 minutes, and incubated with appropriate secondary antibodies on the rocking bed for 1 h at room temperature. Antibody binding was detected using enhanced chemiluminescence (ECL) Pro-Light horseradish peroxidase (HRP) kit (Tiangen Biotechnology Company, Beijing, China) and photos were taken by means of FluorChem FC2 imaging system (Nature Gene, America). Signals were quantified using CMIAS-II image analysis system (Beijing University of Aeronautics & Astronautics, Beijing, China) and compared with the integral optical density (IOD) values.

### 2.7. Statistical Analysis

The data were analyzed by one way ANOVA using of the statistical package for the social sciences (SPSS) 13.0 statistical software. The data were described using mean and standard deviation $\left(\overset{-}{X}\pm s\right)$. Comparing with control group, ∗ was *P* < 0.05, ∗∗ was *P* < 0.01; comparing with neutron irradiation group, # was *P* < 0.05, ## was *P* < 0.01. Values of *P* < 0.05 were considered statistically significant.

## 3. Results

### 3.1. Morphologic Changes of IEC-6

**Figure 1:**
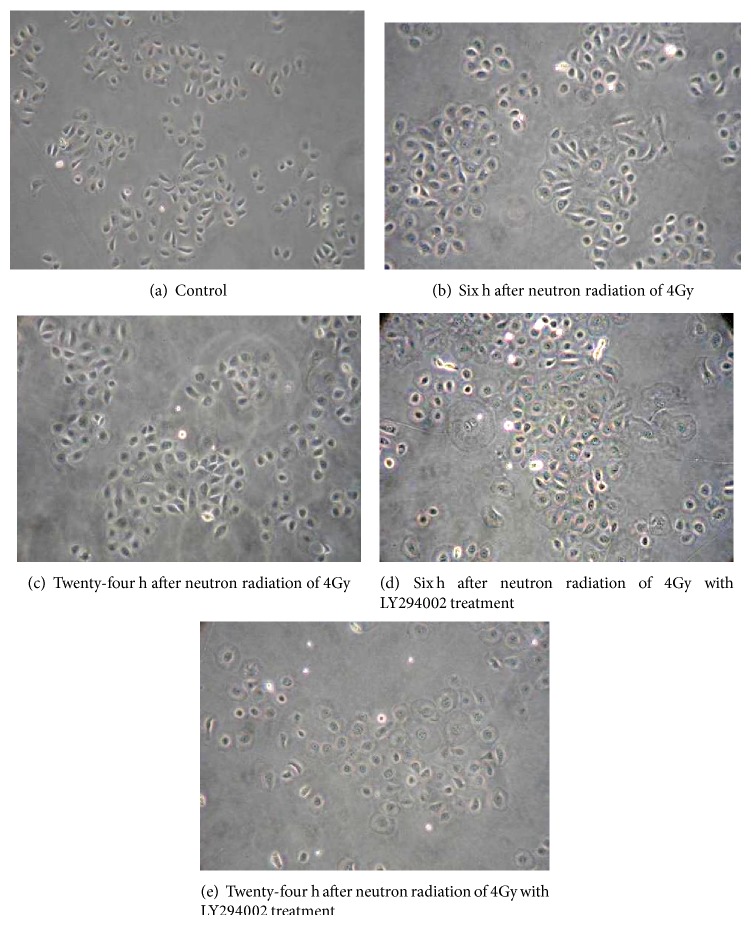
The changes of IEC-6 appearance (IPCM, magnification of 200x). (a) Control, the IEC-6 cells looked like applanatus polygon or fusiform shape and aggregated together shape of chrysanthemum thyse appearance. (b) Six h after neutron radiation of 4Gy, the IEC-6 cells swelled and became approximately round shaped and lots of dead cells floated on the culture solution. (c) Twenty-four h after neutron radiation of 4Gy, the cells were injured more seriously than those of 6 h after neutron irradiation. ((d), (e)) Neutron irradiation of 4Gy with LY294002 treatment group cells, the cells were injured more seriously than those of the neutron irradiation group.

The control group IEC-6 cells grew side by side tightly adhering to the culture flask. The IEC-6 cells aggregated together and looked like cluster or chrysanthemum thyse appearance. The IEC-6 cells looked like applanate polygon or fusiform shape (Figure 1(a)). At 6 h and 24 h after neutron irradiation of 4Gy, the IEC-6 cells swelled and became approximately round shaped and lots of dead cells floated on the culture solution (Figures 1(b) and 1(c)). The LY294002 treatment group IEC-6 cells were injured more seriously than neutron irradiation group (Figures 1(d) and 1(e)).

### 3.2. Proliferation Ability of IEC-6

**Figure 2:**
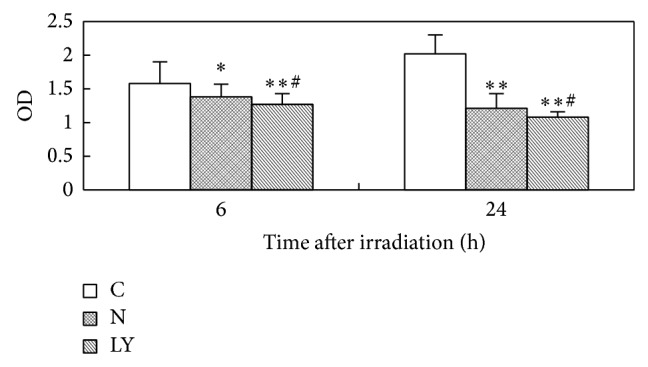
The proliferation ability changes of IEC-6 cells after neutron irradiation of 4Gy and using of LY294002 (C: control group; N: neutron irradiation group; LY: neutron irradiation of 4Gy with LY294002 treatment group).

The proliferation ability of the control group IEC-6 cells was increased gradually in 24 h. The proliferation ability of the neutron irradiation group IEC-6 cells was decreased obviously in 24 h of neutron irradiation of 4Gy. The descent tendency of the LY294002 treatment group cells was more obvious than that of the neutron irradiation group.  Figure 2 showed the results of statistical analysis.

### 3.3. Apoptosis and Necrosis Rates of IEC-6

**Figure 3:**
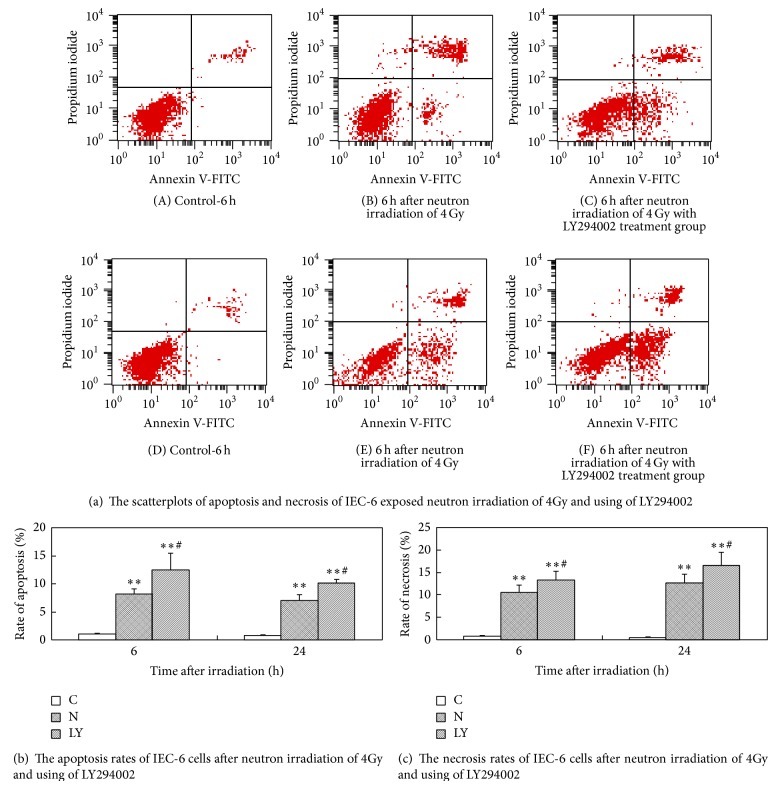
The apoptosis and necrosis rates of IEC-6 cells after neutron irradiation of 4Gy and using of LY294002 (C: control group; N: neutron irradiation group; LY: neutron irradiation of 4Gy with LY294002 treatment group).

The apoptosis and necrosis rates of the IEC-6 cells were increased obviously at 6 h and 24 h after neutron irradiation of 4Gy. The apoptosis rates of the IEC-6 cells were the peak value at 6 h after neutron irradiation, while most of the cells appeared necrosis at 24 h after neutron irradiation. The apoptosis and necrosis rates of the LY294002 treatment group IEC-6 cells were higher than those of the neutron irradiation group cells.  Figure 3(a) (A, B, C, D, E, F) showed the scatterplot of apoptosis and necrosis of IEC-6 exposed to neutron irradiation of 4Gy and treated by LY294002. Figures 3(b) and 3(c) showed the results of statistical analysis.

### 3.4. Expressions of the Key Signaling Molecule of NF-*κ*B Signaling Pathway in IEC-6 (Figure 4)

#### 3.4.1. NF-*κ*B (p65) and Phosphor-NF-*κ*B

The expressions of NF-*κ*B (p65) in the IEC-6 cells were upregulation at 6 h and 24 h after neutron irradiation of 4Gy. The expressions of phosphor-NF-*κ*B in the IEC-6 cells were upregulation from 30 min to 6 h (reaching peak at 6 h) after neutron irradiation of 4Gy, while no expressions were assayed at 12 h and 24 h after neutron irradiation of 4Gy. The expressions of NF-*κ*B (p65) and phosphor-NF-*κ*B were inhibited by LY294002.

#### 3.4.2. IKK*α*, IKK*β*, and Phosphor-IKK*α*/*β*

The expressions of IKK*α* and IKK*β* in the IEC-6 cells were upregulation at 6 h and 24 h after neutron irradiation of 4Gy. There were two subunits in IKKs (*α*/*β*), so two electrophoresis strips were assayed for phosphor-IKK*α*/*β*. The expressions of phosphor-IKK*α*/*β* in the IEC-6 cells were upregulation from 30 min to 6 h (reaching peak at 6 h) after neutron irradiation of 4Gy, while no expressions were assayed at 12 h and 24 h after neutron irradiation of 4Gy. The expressions of IKK*α*, IKK*β*, and phosphor-IKK*α*/*β* were inhibited by LY294002.

#### 3.4.3. I*κ*B*α* and Phosphor-I*κ*B*α*

The expressions of I*κ*B*α* in the IEC-6 cells were downregulation at 6 h and 24 h after neutron irradiation of 4Gy. No expressions of phosphor-I*κ*B*α* were assayed at 30 min and 2 h after neutron irradiation of 4Gy, while the expressions of phosphor-I*κ*B*α* reached peak at 6 h after neutron irradiation of 4Gy. No expressions were assayed at 12 h and 24 h after neutron irradiation of 4Gy. The expressions of I*κ*B*α* and phosphor-I*κ*B*α* were increased by LY294002.

### 3.5. Expressions of PI3K and Phosphor-PI3K, Akt and Phosphor-Akt in IEC-6

**Figure 5:**
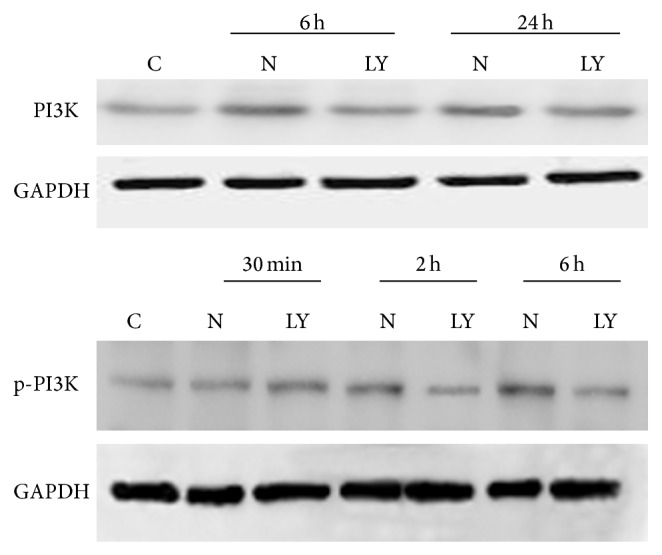
The expressions of PI3K and p-PI3K in IEC-6 cells after neutron irradiation of 4Gy and using of LY294002 (C: control group; N: neutron irradiation group; LY: neutron irradiation of 4Gy with LY294002 treatment group).

**Figure 6:**
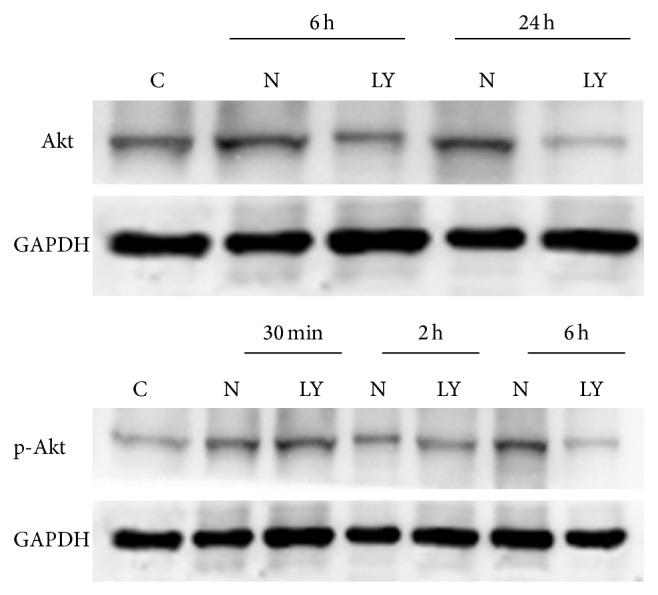
The expressions of Akt and p-Akt in IEC-6 cells after neutron irradiation of 4Gy and using of LY294002 (C: control group; N: neutron irradiation group; LY: neutron irradiation of 4Gy with LY294002 treatment group).

The expressions of PI3K in the IEC-6 cells were upregulation at 6 h and 24 h after neutron irradiation of 4Gy. The expressions of phosphor-PI3K and phosphor-Akt in the IEC-6 cells were upregulation from 30 min to 6 h (reaching peak at 6 h) after neutron irradiation of 4Gy, while no expressions were assayed at 12 h and 24 h after neutron irradiation of 4Gy. The expressions of PI3K and phosphor-PI3K, Akt and phosphor-Akt were inhibited by LY294002. Figures 5 and 6 showed the results of Western blotting.

## 4. Discussion

As we all know, neutron is high lineal energy transfer (LET) ionizing irradiation which can cause bodies more severe damage than *γ* rays. The intestine is highly sensitive to neutron irradiation, severely injured by neutron irradiation, and hard to recover. Unfortunately, there is still no effective therapeutic measure so far. IEC-6 cells coming from the normal SD rat jejunum crypt epithelial cells can reflect the characteristics of the intestine epithelial cells and can be generally used in the study on the in vitro model of intestine diseases. IEC-6 cells are highly sensitive to ionizing radiation. When the cell lines were irradiated by ionizing radiation, their proliferation activity decreased seriously and there was obvious dose-effect relationship [6]. Therefore, in this study, intestinal epithelial cell (IEC) model was made which was injured by neutron irradiation of 4Gy. We would investigate NF-*κ*B signaling pathway in the regulation of IEC damaged by neutron irradiation. This could be sought to elucidate the molecular mechanism of neutron irradiation-induced intestinal injury, which might help to find new potential therapies. At the same time, we would study how PI3K (the upstream signaling molecule of NF-*κ*B) regulates the NF-*κ*B signaling pathway.

NF-*κ*B is an important nuclear factor which resides in cells widely. It is composed of p65 and p50 which are the two important subunits. NF-*κ*B combines I*κ*B (repressor of NF-*κ*B) which forms an unreactive trimer in the quiescent condition cytoplasm. Lots of extracellular harmful factors such as tumour necrosis factor-*α*, lymphotoxin-*β*, and irradiation can activate I*κ*B kinase (IKK) which can lead to I*κ*B phosphorylation and ubiquitination, degradation. At the same time, as soon as NF-*κ*Bp50/65 subunits are liberated in the cytoplasm, NF-*κ*B is then free to translocate to the nucleus and bind DNA leading to the activation of target genes [6–8]. In the previous reports, many studies showed NF-*κ*B regulates the target genes correlation with immune and inflammatory reaction. However, now, more and more studies showed NF-*κ*B also could regulate some genes correlation with cell proliferation, apoptosis, and differentiation [9, 10].

PI3K is the upstream molecule of NF-*κ*B signaling pathway. NF-*κ*B signaling pathway activation depends on PI3K/Akt activating in some cell injury models. Activating NF-*κ*B signaling pathway can negatively regulate apoptosis and improve the cells proliferation [11, 12]. LY294002 is the classic inhibitor of PI3K; it can specifically inhibit the activation of PI3K which can lead to inhibiting the activation of PI3K/Akt signaling pathway [13]. Some extracellular harmful factors such as virus, interferon, and irradiation can activate PI3K/Akt signaling pathway which can induce NF-*κ*B signaling pathway activation [14, 15], while up to now, there are no studies on the function mechanism of NF-*κ*B signaling pathway in intestinal epithelial cell injured by neutron irradiation and how PI3K/Akt signaling pathway regulates NF-*κ*B signaling pathway.

In this study, we found IEC-6 cell lines showed serious apoptosis and necrosis and cell proliferation activity depression after being irradiated by neutron of 4Gy, while the IEC-6 cells treated by LY294002 (inhibitor of PI3K) and exposed to neutron irradiation of 4Gy showed higher apoptosis and necrosis rates than the neutron irradiation group cells. The expression of the critical signaling molecules of NF-*κ*B signaling pathway in the IEC-6 cells such as NF-*κ*B (p65) and IKK*α*/*β* was upregulated after being irradiated by neutron of 4Gy, while I*κ*B*α* (the repressor of NF-*κ*B) was downregulated. The expression of PI3K in the IEC-6 cell lines was upregulated after neutron irradiation of 4Gy, while using LY294002 could inhibit the expression of PI3K. We also found LY294002 could downregulate the expression of NF-*κ*B (p65) and IKK*α*/*β*. These results showed neutron irradiation could activate the NF-*κ*B signaling pathway in IEC-6 cells. Moreover, PI3K could positively regulate NF-*κ*B signaling pathway in IEC-6 cells, while using the inhibitor of PI3K could also inhibit the activation of NF-*κ*B pathway which led to aggravating the IEC-6 cells damage.

In this study, we explored the function of NF-*κ*B signaling pathway in the IEC-6 injured by neutron irradiation. We found neutron irradiation could activate NF-*κ*B signaling pathway and PI3K positively regulated NF-*κ*B signaling pathway in IEC-6 cells, while LY294002, inhibitor of PI3K, could also inhibit the activation of NF-*κ*B signaling pathway in IEC-6 cells irradiated by neutron of 4Gy which led to aggravating the IEC-6 cells injury. On the contrary, this result indicated that activating NF-*κ*B signaling pathway could protect the IEC-6 cells injured by neutron irradiation. This discovery provided theoretical evidence and foundation for elucidating the molecule mechanism of intestine damaged by neutron irradiation and finding new therapy target.

## 5. Conclusions

Results of this study suggest that IEC-6 cells were obviously damaged and induced serious apoptosis and necrosis by neutron irradiation of 4Gy; the NF-*κ*B signaling pathway in IEC-6 was activated by neutron irradiation which could protect IEC-6 against injuries by neutron irradiation; LY294002 could inhibit the proliferation activity of IEC-6 cells.

## Figures and Tables

**Figure 4:**
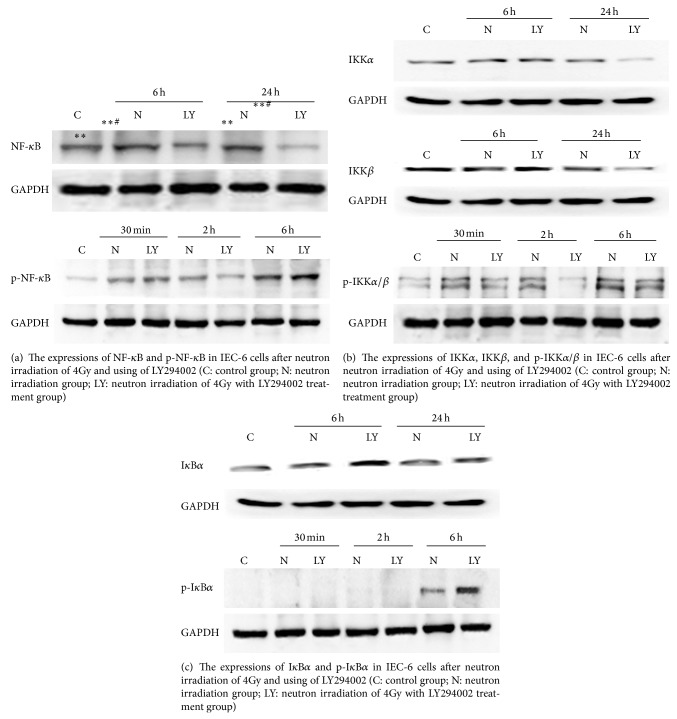
The expressions of key molecules of NF-*κ*B signaling pathway in IEC-6 cell lines after neutron irradiation of 4Gy and using of LY294002 (C: control group; N: neutron irradiation group; LY: neutron irradiation of 4Gy with LY294002 treatment group).
